# Visualization of thermal damage using ^68^ Ga-FAPI-PET/CT after pulmonary vein isolation

**DOI:** 10.1007/s00259-021-05612-9

**Published:** 2021-11-15

**Authors:** Jana Kupusovic, Lukas Kessler, Stephan G. Nekolla, Lisa Riesinger, Manuel M. Weber, Justin Ferdinandus, Simon Kochhäuser, Tienush Rassaf, Reza Wakili, Christoph Rischpler, Johannes Siebermair

**Affiliations:** 1 Department of Cardiology and Vascular Medicine, West German Heart and Vascular Center Essen, University of Duisburg-Essen, Hufelandstrasse 55, 45147 Essen, Germany; 2 Department of Nuclear Medicine, University Hospital Essen, University of Duisburg-Essen, Hufelandstrasse 55, 45147 Essen, Germany; 3 Department of Nuclear Medicine, Klinikum Rechts Der Isar, Technical University Munich, Munich, Germany; 4 DZHK (German Centre for Cardiovascular Research), Partner Site Munich Heart Alliance, Munich, Germany

**Keywords:** FAPI, PET, Fibroblast activation, Pulmonary vein isolation, Atrial fibrillation, Catheter ablation, Cardiovascular imaging

## Abstract

**Purpose:**

^68^ Ga-fibroblast-activation protein inhibitor (FAPI) positron emission tomography (PET) is a novel technique targeting FAP-alpha. This protein is expressed by activated fibroblasts which are the main contributors to tissue remodeling. The aim of this proof-of-concept study was to assess ^68^ Ga-FAPI uptake in the pulmonary vein (PV) region of the left atrium after pulmonary vein isolation (PVI) with cryoballoon ablation (CBA) and radiofrequency (RFA) as a surrogate for thermal damage.

**Methods:**

Twelve PVI patients (5 RFA, 7 CBA) underwent ^68^ Ga-FAPI-PET 20.5 ± 12.8 days after PVI. Five patients without atrial fibrillation or previous ablation served as controls. Standardized uptake values of localized tracer uptake were calculated.

**Results:**

Focal FAPI uptake around the PVs was observed in 10/12 (83.3%) PVI patients, no uptake was observed in 2 PVI patients and all controls. Patients after PVI had higher FAPI uptake in PVs compared to controls (SUV_max_: 4.3 ± 2.2 vs. 1.6 ± 0.2, *p* < 0.01; SUV_peak_: 2.9 ± 1.4 vs. 1.3 ± 0.2, *p* < 0.01). All CBA patients had an intense uptake, while in the RFA, group 2 (40%), 1 (20%), and 2 (40%) patients had an intense, moderate, and no uptake, respectively. We observed higher uptake values (SUV_peak_) in CBA compared to RFA patients (4.4 ± 1.5 vs. 2.5 ± 0.8, *p* = 0.02).

**Conclusion:**

We demonstrate in-vivo visualization of ^68^ Ga-FAPI uptake as a surrogate for fibroblast activation after PVI. CBA seems to cause more pronounced fibroblast activation following tissue injury than RFA. Future studies are warranted to assess if this modality can contribute to a better understanding of the mechanisms of AF recurrence after PVI by lesion creation and gap assessment.

**Supplementary Information:**

The online version contains supplementary material available at 10.1007/s00259-021-05612-9.

## Introduction

Pulmonary vein isolation (PVI) has become the interventional cornerstone therapy of atrial fibrillation (AF) [1]. The procedural endpoint of PVI is a complete electrical block between the PVs and the left atrium (LA) preventing conduction of electrical triggers from the PVs to the atria, which efficiently reduces AF recurrence in both paroxysmal and persistent AF [2]. The most established PVI techniques include radiofrequency (RFA) and cryoballoon ablation (CBA), both aiming to create a circumferential transmural ablation lesion between the LA and the PVs. Recurrence rates after PVI are high, ranging from 25 to 40% within a year after the procedure [3] which can (at least partly) be attributed to incomplete and/or nondurable ablation lesions [4, 5]. While RFA, as well as CBA, demonstrated comparable efficacy and safety outcomes [6] a few studies mainly based on laboratory results suggested a higher extent of myocardial injury after CBA compared to RFA [7–9]. Novel techniques to visualize the ablation sets following thermal damage in PVI and to assess potential differences of lesion characteristics between CBA and RFA procedures seem crucial for a better understanding of the mechanisms behind AF recurrence [5, 10].

PET imaging with FAP inhibitor (FAPI) radiotracers has emerged as a new tool in the visualization of fibroblast activation. This serinprotease is specifically expressed by activated fibroblasts, e.g., in chronic inflammation, tumor progression, wound healing, or fibrosis [11]. This imaging technique may offer new insights in visualizing myocardial damage after PVI. The aim of this proof-of-concept study was to assess ^68^ Ga-FAPI uptake in the antral PV region of the LA after PVI using either cryo- or radiofrequency energy as a potential surrogate for local tissue response following thermal damage.

## Material and methods

### Patient population

Twelve patients who had undergone ^68^ Ga-FAPI-PET after PVI were included. Five patients matched for age, sex, and left ventricular ejection fraction without a history of AF, any cardiac comorbidity or previous ablation having undergone ^68^ Ga-FAPI-PET for tumor staging served as controls. Patients treated with cardio-toxic drugs (e.g., anthracyclines or immune checkpoint inhibitors) or external-beam radiation therapy to the chest were excluded to avoid distortion of myocardial FAPI uptake due to confounders [12]. We assessed clinical baseline characteristics, imaging parameters from echocardiography, and procedural PVI data. The investigations were conducted in accordance with the Declaration of Helsinki and national regulations. All patients gave written informed consent to undergo FAPI PET/CT following the regulations of the German Pharmaceuticals Act §13(2b). Retrospective analysis of PET/CTs and clinical data was approved by the local ethics committee for the purpose of the present study (permit no. 20–9777-BO).

### Pulmonary vein isolation techniques

Circumferential CBA was performed by a second-generation cryoballoon after a transseptal puncture. One to three cryo-energy applications, each 240 s in duration, were intended for each PV. In RFA procedures, operators used a point-by-point approach with an open-irrigated single tip catheter (ThermoCool SurroundFlow®, Biosense Webster) to create a contiguous circular lesion around each PV antrum.

### Radiotracer synthesis

A detailed description of ^68^ Ga-FAPI-46 synthesis is provided in the online supplement.

### Image acquisition

PET scans were performed on a PET/computer tomography (CT) system (Biograph Vision, Siemens Healthineers, Erlangen, Germany). The injected activity of ^68^ Ga-FAPI was 119 ± 22 MBq [80;158 MBq] resulting in a calculated mean effective dose of 1.95 ± 0.4 mSv. ECG-gated cardiac PET imaging was performed approximately 60 min p.i. PET images were reconstructed using an ordered subset expectation maximization (OSEM) algorithm, including time-of-flight information, with 4 iterations and 8 subsets. A Gaussian filter kernel with a full width at a half maximum of 4 mm was used for post-reconstruction filtering. Low-dose CT was performed for attenuation correction (30 mAs, 120 keV, 512 × 512 matrix, 3 mm slice thickness). In addition, a CT angiography (CTA) was performed (30–40 mL iomeprol; 400 mg iodine per milliliter; Iomeron 400; Bracco, Milan, Italy) with the following parameters: spiral mode, 0.6 s gantry rotation; collimation, 64 × 0.6 mm; pitch, 1.375:1; section thickness, 0.6 mm; reconstruction interval, 0.5 mm; tube voltage: 120 kV; current intensity: 300 mA.

### Image evaluation

PET data were analyzed by two nuclear medicine specialists (CR and LK) on a consensus decision. Tracer uptake was visually rated as “intense,” “moderate,” or “absent” if uptake was clearly higher, slightly higher, or comparable to blood pool in RA, respectively. Tracer uptake was quantified as maximum (SUV_max_) and peak (SUV_peak_) standardized uptake values from static images 60 min after tracer injection. For this purpose, a region grow algorithm at the PV ostia with a threshold of 40% of the maximum uptake was performed (Syngo.via software; Siemens Healthineers, Erlangen, Germany) and a volume-of-interest for each PV ostium was defined. Background (bloodpool, right atrium) was quantified using a circular 1 cm diameter sphere.

### Statistical analysis

Statistical analysis was performed using GraphPad Prism (version 8.4.2; GraphPad Software, San Diego, California USA), with quantitative values expressed as mean ± standard deviation or median and range where appropriate. Comparison of non-parametric data was performed using a Mann–Whitney *U* test or Kruskal–Wallis test for multiple comparisons. All tests were performed two-sided and a *p*-value < 0.05 was considered to indicate statistical significance.

## Results

### Patient and procedural characteristics

Patient characteristics are presented in Table 1. The PVI cohort consisted of 12 patients (83.3% male), with a mean age of 60.8 ± 11.3 years, thereof 7/12 (58.3%) with paroxysmal AF. Five patients (41.7%) underwent RFA and 7 patients (58.3%) CBA. The median time between PVI and PET/CT was 21 [range 3–48] days.

**Table 1:** Patient characteristics

	PVI group, *n* = 12	Controls, *n* = 5	*P*-value
Male sex, *n* (%)	10 (83.3)	4 (80)	0.87
Age at scan, years	60.8 ± 11.3	58.4 ± 3.8	0.57
LVEF, %	54.8 ± 7.3	57.2 ± 7.6	0.72
Paroxysmal AF, *n* (%)	7 (58.3)	0 (0)	< 0.01
Persistent AF, *n* (%)	5 (41.7)	0 (0)	< 0.01
CHF, *n* (%)	4 (33.3)	1 (20)	0.58
LAVI, mL	37.8 ± 19.9	25.2 ± 3.4	0.33
BMI, kg/m^2^	28.7 ± 5.6	25.8 ± 4.6	0.33
Cardiovascular risk factors			
Arterial hypertension, *n* (%)	10 (83.3)	3 (60)	0.30
Hyperlipoproteinemia, *n* (%)	4 (33.3)	1 (20)	0.58
Tabacco use, *n* (%)	4 (33.3)	2 (40)	0.79
Diabetes, *n* (%)	1 (8.3)	1 (20)	0.50
History of stroke/TIA, *n* (%)	0 (0%)	0 (0%)	1.00
CHA_2_DS_2_VASc score, points	2.3 ± 1.5	NA	

*LVEF*, left ventricular ejection fraction; *AF*, atrial fibrillation; *CHF*, chronic heart failure; *LAVI*, left atrial volume index; *BMI*, body mass index; *TIA*, transient ischemic attack; *RF*, radiofrequency ablation; *PVI*, pulmonary vein isolation; *CBA*, cryoballoon ablation

### Imaging results

#### PVI patients vs. controls

**Fig. 1:**
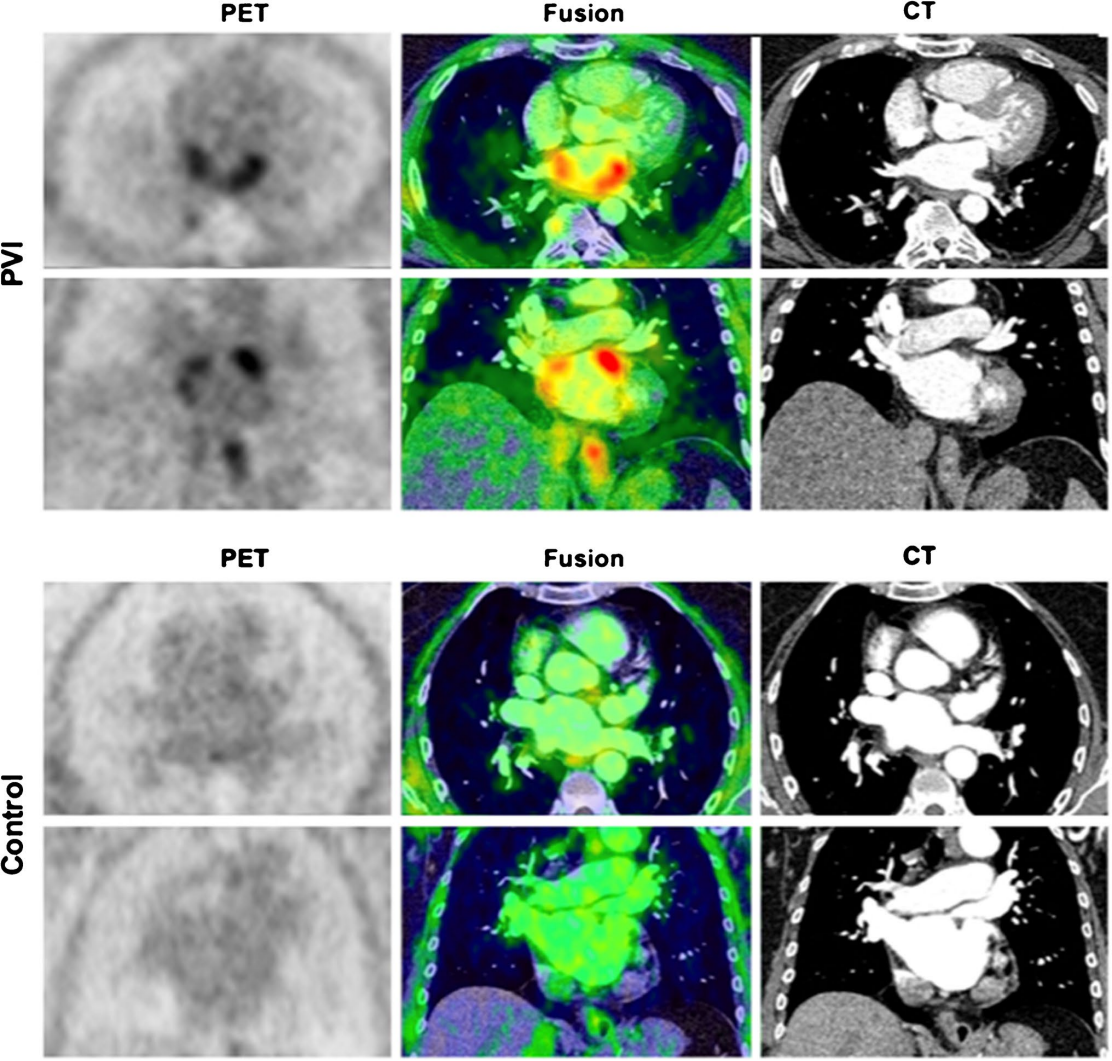
Localized FAPI uptake in a PVI patient vs. control. Upper row (PVI): example of a distinct focal visual uptake in PV antra after PVI; lower row (control): no visual uptake in a patient having undergone PET imaging for tumor staging; PET (left panel), PET/CT fusion (middle panel) and CT (right panel). PV, pulmonary veins; PVI, pulmonary vein isolation; PET, positron emission tomography; CT, computed tomography

**Fig. 2:**
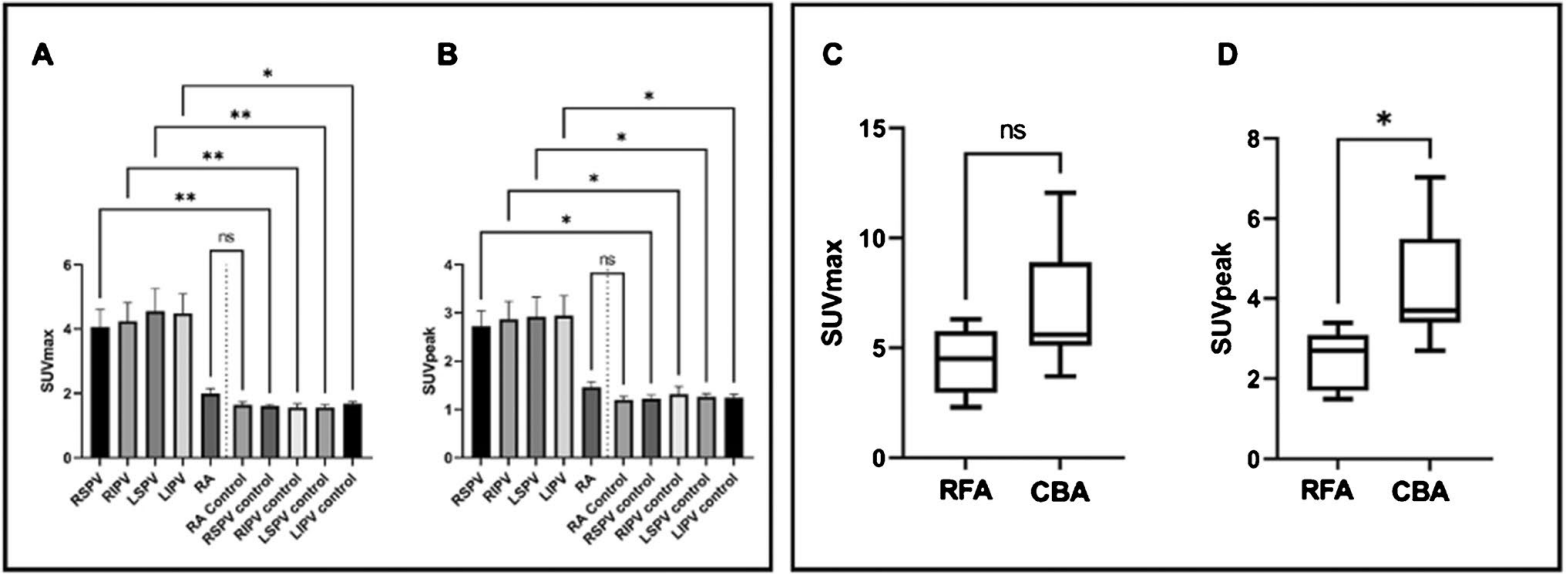
Difference in specific PET parameters in PVI vs. controls and RFA vs. CBA ablations. Left panel: The distribution of the specific PET parameters SUV_max_ (A) and SUV_peak_ (B) between uptake at PV antra in ablated (first 4 columns) and ablation-naïve individuals (controls) is depicted, demonstrating quantitatively significantly higher tracer uptake in PVI patients. RA uptake in PVI and ablation-naïve individuals was comparable highlighting the reliability of the results. Right panel: the quantitative differences (SUV_max_) between RFA and CBA (C) with a trend of higher uptake for CBA compared to RFA patients; in (D) for SUV_peak_, where significantly higher tracer uptake for CBA patients was observed. RFA, radiofrequency ablation; CBA, cryoballoon ablation; RSPV, right superior pulmonary vein; RIPV, right inferior pulmonary vein; LSPV, left superior pulmonary vein; LIPV, left inferior pulmonary vein; RA, right atrium; *, *p* ≤ 0.05; **, *p* ≤ 0.01; ns, non-significant

Of the 12 PVI patients, 9 (75%) and 1 (8.3%) patients had an intense and moderate visual uptake in PV ostia, respectively, while no uptake was observed in 2 PVI patients and all controls. Examples of intense uptake in a PVI patient vs. no uptake in a control are shown in Fig. 1. PVI patients revealed a significant higher FAPI uptake in PVs compared to controls for both SUV_max_ (4.3 ± 2.2 vs. 1.6 ± 0.2, *p* < 0.01) and SUV_peak_ (2.9 ± 1.4 vs. 1.3 ± 0.2, *p* < 0.01). There was no difference in background RA tracer uptake between the two cohorts. Quantitative differences between PET parameters of the PVI and control group are presented in Fig. 2A and B.

#### Comparison of the specific ablation techniques (RFA vs. CBA)

All CBA patients had an intense uptake, while in the RFA group 2 (40%), 1 (20%), and 2 (40%) patients had an intense, moderate, or no visual uptake, respectively. Baseline and procedural characteristics in RFA vs. CBA patients are provided in Supplemental Table 1. Further, we observed significantly higher values of SUV_peak_ in CBA compared to RFA patients (4.4 ± 1.5 vs. 2.5 ± 0.8, *p* = 0.02) and a strong trend towards higher values of SUV_max_ for CBA patients (6.8 ± 2.8 vs. 4.4 ± 1.5, *p* = 0.11). PET-derived parameters of RFA and CBA patients are presented in Fig. 2C and D.

## Discussion

This retrospective study is the first assessing FAPI uptake as a surrogate for fibroblast activation in patients after PVI showing the capability of this modality to visualize myocardial damage after catheter ablation. We observed increased tracer uptake in the majority of PVI patients compared to no uptake in the control group clearly indicating specific fibroblast activation following thermal injury. With respect to quantitative measurements, we observed a significantly higher tracer uptake in the antra of PVI patients compared to the control group. Tracer uptake in CBA procedures was higher than in RF procedures pointing at more pronounced fibroblast activation by the single-shot device.

### PVI patients vs. controls

Our results indicate that tracer uptake in PVI patients markedly exceeded the uptake in controls both qualitatively (i.e., visually) and quantitatively. Quantitative measures SUV_max_ and SUV_peak_ demonstrate the markedly higher uptake at the antra of all 4 PVs in ablated patients vs. controls pointing at the ability of this novel technique to show fibroblast activation as a surrogate for thermal damage following PVI. With respect to the 2 PVI patients (both RFA group) without tracer uptake, we hypothesize that imaging was done too early in one patient (3 days after PVI), as the peak FAPI uptake is expected more than 6 days following myocardial damage as derived from imaging and histopathologic studies on myocardial infarction [13]. Our post hoc derived analysis within the ablation subgroups (*n* = 5 RFA and *n* = 7 CBA patients) failed to correlate quantitative tracer uptake with the time interval between ablation and PET (Supplemental Table S2). As we cannot rule out the bias of the very small sample size on this result, there is an urgent need for studies assessing the potential impact of the timepoint of PET imaging after PVI on fibroblast activation. The second reason for non-detectable uptake in these two RF patients may depend on the applied ablation method.

### Cryoablation vs. radiofrequency ablation

When stratifying FAPI uptake for the applied PVI method, we observed a pronounced tracer uptake in all CBA patients and in only 3/5 (60%) RFA patients. This finding is in line with previous reports demonstrating that the level of biomarkers reflecting myocardial injury is more pronounced in PVI with CBA than RFA procedures, which has been confirmed by MRI studies suggesting a wider, more extended tissue damage after CBA compared to RFA [7–9, 14].

### Clinical implications

In the past, magnetic resonance imaging (MRI) has been investigated with respect to its capacity to visualize cardiac ablation lesions using late gadolinium enhancement (LGE) [15–17]. These studies have shown the general ability of LGE imaging to assess ablation lesions following thermal damage, nevertheless have failed to reliably predict sites of electrical re-conduction following PVI [10]. As electrical re-conduction is suggested to constitute a crucial factor for AF recurrence, this method has important limitations [4]; further no controlled study so far could reliably demonstrate a correlation of MRI-derived ablation lesions and mid-/long-term outcome with respect to AF recurrence pointing at the actual limitations of this method. From a mechanistic point of view, it could be assumed that LGE imaging targeting the late diffusion of contrast in the extracellular volume as a surrogate for scarred tissue after ablation potentially cannot differentiate between pre-existing fibrosis/scar and ablation damage [17]. These issues together with the important methodical limitations of low reproducibility of LGE assessment in inexperienced centers point to the need for other new noninvasive imaging methods [18].

Our newly developed method for the functional assessment of local damage by PVI seems promising for a better understanding of the mechanisms behind AF recurrence. This is especially relevant as besides the established thermal ablation systems (cryo- and RFA ablation) new energy sources for PVI (pulsed-field ablation, PFA) are arising which hold promise for complete electrical isolation by nonthermal electroporation. The continuously improving spatio-temporal resolution of modern PET/CT scanners will potentially provide the opportunity to assess gaps in ablation sets in the future by functional assessment of fibroblast activation which can potentially be further improved by PET/MRI approaches. This could help to better understand the impact of incomplete PV isolation on AF recurrence. [5] ^68^ Ga-FAPI-PET is a promising imaging tool that could help to fill in these “gaps in knowledge”; however, larger, prospective studies and validation of PV reconnection by invasive EP studies are needed to further explore this application.

### Limitations

This retrospective study has a few limitations. We investigated the novel FAPI imaging in a small cohort after PVI. Further, we are not able to provide histological validation data on tissue samples. Lastly, we did not perform FAPI PET/CT before ablation making it impossible to discriminate between uptake for thermal lesion and potentially pre-existing local fibroblast activation.

## Conclusion

According to these preliminary results, ^68^ Ga-FAPI uptake as a surrogate for fibroblast activation in patients after PVI can be visualized and assessed using PET/CT. PVI with CBA seems to cause more intense fibroblast activation and tissue injury. Future studies are warranted to assess if this modality can contribute to a better understanding of the mechanisms of AF recurrences after PVI by thermal lesion assessment as fibroblast activation constitutes a promising target for imaging studies on thermal and nonthermal ablation methods, ideally in combination with (invasive) electrophysiological confirmation of ablation lesions.

## Supplementary Information

Below is the link to the electronic supplementary material.

Supplementary file1 (DOCX 22 KB)

## Data Availability

The data that support the findings of this study are available from the corresponding author, [CR], upon reasonable request.
