# Supplementary material for: Visualization of thermal damage using 68 Ga-FAPI-PET/CT after pulmonary vein isolation
Source: Eur J Nucl Med Mol Imaging. 2021 Nov 15;49(5):1553–9. doi: 10.1007/s00259-021-05612-9 (PMC8940837; doi:10.1007/s00259-021-05612-9)
Supplement: Supplementary file 1 — Supplementary file1 (DOCX 22 KB) [file 259_2021_5612_MOESM1_ESM.docx]

**SUPPLEMENTAL MATERIAL**

**Radiotracer synthesis**

The synthesis of ^68^Ga-FAPI-46 was performed on a Scintomics GRP 4V module with a module system (Scintomics GmbH, Fürstenfeldbruck, Germany) connected to two ^68^Ge/^68^Ga generators (1850 MBq, GalliaPharm®, Eckert and Ziegler, Berlin, Germany). Before the automated synthesis, the reaction vial is pre-loaded with 75 µg FAPI (ABX, Radeberg) and 3 mg of ascorbic acid dissolved in 1.6 mL of sodium acetate buffer. The elution of the generators with 0.1 N HCl is fully automatically performed using a GMP-grade cassette system which is controlled by a modified [^68^Ga]Ga-HBED-cc-PSMA program sequence. The solution is passed through a cation exchange cartridge (type PS-H+). The material on the cartridge binds ^68^Ga, whereas impurities like ^68^Ge and other metal cations remain in the solution. Subsequently, ^68^Ga is eluted from the PS-H+ cartridge into the reaction vial using eluent solution. For radio-labeling, the reaction mixture is heated to 95 °C for 20 min. For purification the reaction mixture is passed through a Sep-Pak® Light 18C cartridge and the retained crude ^68^Ga-FAPI is washed with water for injection. ^68^Ga-FAPI is extracted (from the Sep-Pak® Light ^18^C cartridge) using ethanol/water (1:1). The drug substance ^68^Ga-FAPI is transferred into the bulk vial through a sterile filter and formulated with phosphate buffered saline. The quality control procedures included RP-HPLC, ITLC (colloid), pH, endotoxin and sterility testing and ^68^Ge breakthrough measurement.

**Supplementary table 1.** Patient characteristics and ablation data, stratified for RFA and CBA

|  | | **RFA, n=5** | **CBA, n=7** |
| --- | --- | --- | --- |
| Male sex, n (%) | | 3 (60%) | 7 (100%) |
| Age at scan, years | | 56.0±10.0 | 64.1±11.5 |
| LVEF, % | | 54.2±9.4 | 55.1±6.2 |
| Paroxysmal AF, n (%) | | 4 (80) | 3 (42.9) |
| CHF, n (%) | | 1 (20) | 3 (42.9) |
| CAD, n (%) | | 1 (20) | 3 (42.9) |
| LAVI, mL | | 45.4±26.7 | 32.4±13.1 |
| BMI, kg/m² | | 29.1±6.1 | 28.4±5.7 |
| Arterial Hypertension, n (%) | | 4 (80) | 6 (85.7) |
| Hyperlipoproteinemia, n (%) | | 2 (40) | 2 (28.6) |
| Tabaco use, n (%) | | 2 (40) | 2 (28.6) |
| Diabetes, n (%) | | 0 (0) | 1 (14.3) |
| CHA_2_DS_2_VASc score, points | | 2.0±1.4 | 2.4±1.7 |
| Time PVI to Scan, days (median) | | 18.4±13.9 (18) | 22.9±12.8 (21) |
| Total Procedure Time, min | | 260.4±100.9 | 167.1±39.7 |
| LA dwell time, min | | 84.3±20.3 | 55.0±8.4 |
| Radiation Dose, cGy | | 1022.1±370.6 | 1644.7±1355.4 |
| Fluoroscopy Time, min | | 22.9±7.3 | 21.9±5.7 |
| RFA | | | |
|  | Energy Applications, n | 52.8±15.6 |  |
|  | RF Time, min | 54.1±19.5 |  |
| CBA | | | |
|  | Number of Freezes, n |  | 8.0±2.1 |
|  | Total Freeze Time, s |  | 1304.6±531.8 |

RFA, radiofrequency ablation; CBA, cryoablation; LVEF, left ventricular ejection fraction; AF, atrial fibrillation; CHF, chronic heart failure; CAD, coronary artery disease; LAVI, left atrial volume index; BMI, body mass index; PVI, pulmonary vein isolation; LA, left atrium;

**Supplementary table 2.** Correlation of timepoint of imaging and quantitative tracer uptake

|  | | **CBA** | | | | | **RFA** | | |  |
| --- | --- | --- | --- | --- | --- | --- | --- | --- | --- | --- |
|  | **Time interval vs. SUV_peak_** | | | **Time interval vs. SUV_max_** | | **Time interval vs. SUV_peak_** | | | **Time interval vs. SUV_max_** | |
| **Patients (n)** | 7 | | 7 | | 5 | | | 5 | |  |
| **Spearman r** | 0.627 | | 0.551 | | 0.6 | | | 0.257 | |  |
| **P-value (two-tailed)** | 0.104 | | 0.163 | | 0.242 | | | 0.658 | |  |

Abbreviations as in suppl. Table 1.
